# Associations of Dietary Macroelements with Knee Joint Structures, Symptoms, Quality of Life, and Comorbid Conditions in People with Symptomatic Knee Osteoarthritis

**DOI:** 10.3390/nu14173576

**Published:** 2022-08-30

**Authors:** Yan Zhang, Tianyu Chen, Ping Luo, Shengfa Li, Jianwei Zhu, Song Xue, Peihua Cao, Zhaohua Zhu, Jia Li, Xiaoshuai Wang, Anita E. Wluka, Flavia Cicuttini, Guangfeng Ruan, Changhai Ding

**Affiliations:** 1 Clinical Research Centre, Zhujiang Hospital, Southern Medical University, Guangzhou 510000, China; 2 Menzies Institute for Medical Research, University of Tasmania, Hobart, TAS 7000, Australia; 3 Department of Orthopedics, The Third Affiliated Hospital of Southern Medical University, Guangzhou 510000, China; 4 Department of Spinal Surgery, Changsha Hospital of Hunan Normal University, Changsha 410000, China; 5 Department of Orthopedics, Guangzhou First People’s Hospital, School of Medicine, South China University of Technology, Guangzhou 510000, China; 6 Department of Rheumatology and Immunology, Arthritis Research Institute, The First Affiliated Hospital of Anhui Medical University, Hefei 230000, China; 7 Division of Orthopaedic Surgery, Department of Orthopaedics, Nanfang Hospital, Southern Medical University, Guangzhou 510000, China; 8 School of Public Health and Preventive Medicine, Monash University, Melbourne, VIC 3006, Australia; 9 Clinical Research Centre, Guangzhou First People’s Hospital, School of Medicine, South China University of Technology, Guangzhou 510000, China

**Keywords:** osteoarthritis, dietary macroelements, magnesium, potassium, joint structures, symptoms, quality of life, comorbid condition

## Abstract

Background: Osteoarthritis (OA), the most common joint disease in the elderly, has no cure. Macroelements are vital in human health and their relationships with OA are not clear. Clarifying the relationships between macroelements and OA may assist knee OA management. Methods: This study was a post-hoc analysis using data from a two-year randomized controlled trial among 392 participants with knee OA. Dietary macroelements, including calcium, magnesium, potassium, and phosphorus were computed-based on a semi-quantitative food frequency questionnaire at baseline. Knee joint structures (including cartilage volume, cartilage defect, bone marrow lesions, and effusion-synovitis volume), OA symptoms, quality of life, and OA comorbid conditions (including lower limb muscle strength and depressive symptoms) were assessed at baseline and month 24. Western Ontario and McMaster Universities (WOMAC) Index and depressive symptoms were assessed at baseline and months 3, 6, 12, and 24. Quality of life and lower limb muscle strength were assessed at baseline and months 6, 12, and 24. All analyses were conducted using mixed-effects models. Results: Higher dietary magnesium and potassium were associated with fewer OA symptoms, higher quality of life, greater lower limb muscle strength, and fewer depressive symptoms, but not with knee joint structures. Higher dietary calcium and phosphorus was not associated with any of the OA-related outcomes, except that dietary phosphorus was associated with greater lower limb muscle strength. Conclusions: In the longitudinal analyses, higher dietary magnesium and potassium intake are associated with fewer OA symptoms, higher quality of life, and milder comorbid conditions in patients with knee OA, suggesting dietary magnesium and potassium may have beneficial effects on OA and could be used for knee OA management.

## 1. Introduction

Osteoarthritis (OA), the highly prevalent joint-affecting disease, is most frequently seen in the knees [1]. It is characterized by degradation of articular cartilage, synovitis, and changes in subchondral bone, and is a major source of pain and disability, leading to poor quality of life and huge socioeconomic burden [2]. Although age, female gender, high body mass index (BMI), heavy physical workload, and previous knee injuries were identified as risk factors for knee OA, the pathophysiology and etiology of knee OA are not yet completely clear [3]. Currently, there is no disease-modifying treatment for OA [4]. Therefore, exploring modifiable risk factors is warranted for knee OA management.

Comorbidity is present in more than half of OA patients; therefore, considering comorbid conditions is important for a comprehensive understanding of knee OA [5]. Muscle weakness is common in patients with knee or hip OA, with upper leg muscle strength being reported to be approximately 20–40% lower compared with healthy age-matched controls and linked to symptomatic and radiographic progression of the disease [5]. Approximately one-fifth of people with OA experience depressive symptoms, and concomitant depression in OA patients contributes to its increased disease burden and troubles with disease management [6,7].

Macroelements have vital roles in human health [8]. For instance, calcium is essential for bone health, muscle contraction, and normal blood coagulation [8]. Magnesium activates more than 300 enzymes in the body [8]. Potassium regulates the osmotic pressure within the cell and activates some enzymes [8]. Phosphorus is essential for healthy bones, and for energy storage and production [8]. Since current evidence has shown that OA is a whole joint disease or even a systemic disease [2,9], macroelements deficiency affecting multiple tissues or organs may contribute to the pathogenesis of OA. Furthermore, it is widely recognized that nutrition is important in the maintenance of joint health [2]. Therefore, dietary intake of macroelements may be associated with knee OA, and clarification of the relationship between dietary macroelements and knee OA could provide useful information for knee OA management.

Therefore, the aim of this study was to investigate the associations of dietary macroelements, including calcium, magnesium, potassium, and phosphorus, with knee joint structures, symptoms, quality of life, and comorbid conditions in people with symptomatic knee OA.

## 2. Materials and Methods

### 2.1. Study Design, Setting, and Participants

Participants included in this study were from the Vitamin D Effect on Osteoarthritis (VIDEO) study, a multicenter randomized controlled trial of 413 patients aiming to evaluate the effects of vitamin D supplementation on pain and structural changes in symptomatic knee OA patients with vitamin D deficiency [1]. After excluding 21 participants who had no information about diet quality, we included 392 participants (50.51% male) aged between 50 and 79 years (mean 63.26 years) in this post-hoc analysis. The current study used the same inclusion and exclusion criteria as the original trial and treated the study sample as a cohort. In short, patients with symptomatic knee OA for at least six months and serum vitamin D level between 12.5 mol/L and 60 nmol/L were included, and patients with severe radiographic knee OA (Altman’s atlas grade ≥ 3) and severe knee pain (visual analog scale > 80 mm) were excluded [10]. Eligible participants were assessed according to the American College of Rheumatology criteria for clinical knee OA [11]. When both knees met the previously described inclusion and exclusion criteria, the knee with a higher level of pain was set as the study knee [1]. There were five follow-ups in total (baseline, 3-month, 6-month, 12-month, 24-month). Most of the response rates were higher than 80%, with the low limb muscle strength at 24-month having the lowest sample sizes (Supplementary Table S1).

The VIDEO study (clinicaltrials.gov Identifier: NCT01176344) was approved by the Tasmania Health and Human Medical Research Ethics Committee (reference number H1040) and Monash University Human Research Ethics Committee (reference number CF10/1182-2010000616). Written informed consent was obtained from all participants.

### 2.2. Dietary Macroelements Assessments

Dietary intake was assessed at baseline using a semi-quantitative food frequency questionnaire, namely the Dietary Questionnaire for Epidemiological Studies version 2 (DQES v2). The detail of this questionnaire was described in our previous study [12]. Macroelements, including calcium, magnesium, potassium, and phosphorus, were computed based on the DQES v2 using the Australian NUTTAB 95 database [13].

### 2.3. Knee Structures Assessments

Knee joints were scanned at baseline and month 24 using a 1.5 T whole-body magnetic resonance imaging (MRI) unit using a commercial transmit-receive extremity coil. Two image sequences (sagittal T1-weighted fat-saturated spoiled gradient echo and sagittal T2-weighted fat-saturated fast spin echo) were used, the detailed parameters of the two sequences were described in our previous study [1]. Knee structures, including cartilage volume, cartilage defect, bone marrow lesions (BML), and effusion-synovitis volume, were assessed using MRI.

Cartilage volume was measured on T1-weighted MRI using the previously described image processing techniques [14]. As the cartilage plates were isolated by manually drawing disarticulation contours around the cartilage boundaries in a section-by-section manner, tibial cartilage with less workload compared to femoral cartilage was set as the study cartilage. Total cartilage volume was obtained by summing the cartilage volumes of medial tibial, lateral tibial, and patellar sites. The coefficient of variation for the cartilage volume was 2.1% (medial tibia) and 2.2% (lateral tibia) [1].

Cartilage defects were scored from 0 to 4 on T2-weighted images using a modified Outerbridge classification [15] at the medial tibial, medial femoral, lateral tibial, lateral femoral, and patellar sites. Total cartilage defect score was calculated as summing the five subregional cartilage defect scores, and a higher total cartilage defects score indicated a severer overall cartilage defect. The intraclass correlation coefficient (ICC) of the intra-observer reliability for the cartilage defects ranged from 0.77 to 0.94 [1].

BML was scored from 0 to 3 on T2-weighted images using a modified Whole-Organ Magnetic Resonance Imaging Score [16]. For BML scoring, the medial tibia, medial femur, lateral tibia, and lateral femur were divided into three sub-regions (anterior, central, and posterior), and the scores of the three sub-regions were summed to represent each of the tibiofibular compartments. Total BML score was calculated as summing the BML score of the medial tibia, medial femur, lateral tibia, lateral femur, and patella, and a higher total BML score indicated a more severe overall BML. The ICC of the intraobserver reliability for the BML ranged from 0.93 to 0.98 [1].

Effusion-synovitis volume was measured using a previously published methodology [17] on T2-weighted MRI. The volume of effusion-synovitis in the suprapatellar pouch and other cavities was measured and added together to represent the total effusion-synovitis volume of the knee. The ICC of the intraobserver reliability for the effusion-synovitis volume ranged from 0.96 to 0.97 [16].

### 2.4. Knee OA Symptom Assessment

Knee OA symptoms, including knee pain, joint stiffness, and physical dysfunction, were assessed at baseline and months 3, 6, 12, and 24 using the Western Ontario and McMaster Universities (WOMAC) Index [18]. A higher WOMAC score (range: 0–2400) represents a severe OA symptom.

### 2.5. Assessment of Quality of Life

Quality of life was assessed at baseline and months 3, 6, 12, and 24 using the four-dimensional Assessment of Quality of Life (AQoL-4D) [19] which comprises four dimensions of independent living, social relationships, psychological well-being, and physical senses. A higher score of AQoL-4D (range: 0–36) represents a lower quality of life.

### 2.6. Assessment of Comorbid Conditions

Lower limb muscle strength was measured at baseline and months 3, 6, 12, and 24 using dynamometry at the lower limb. This technique simultaneously measures both legs with quadriceps and hip flexors being the mainly measured muscles [1].

Depressive symptoms were assessed at baseline and months 3, 6, 12, and 24 using the 9-items Patient Health Questionnaire (PHQ-9) [20]. A higher score of PHQ-9 (range: 0–27) indicates more severe depressive symptoms.

### 2.7. Assessment of Covariates

Age, sex, BMI, serum 25-hydroxyvitamin D level, energy intake, education, work status, and work type were chosen as covariates for this study. Among these, except sex (male or female), education (Didn’t finish high school; Finished high school; Trade/Apprenticeship; Certificate/Diploma; Bachelor degree or higher), work status (Full-time employed; Part-time/casual employment; Unemployed; Home Duties; Retired; Student; Other), and work type (Manual; Office/Professional; Not applicable) are categorical or ordinal variables, the others were treated as continuous. Age, sex, education, work status, and work type were acquired using self-reported questionnaires. BMI was calculated based on height and weight (weight (kg)/height^2^ (m^2^)), where height was measured to the nearest 0.1 cm (with shoes removed) using a stadiometer (Leicester Height Measure; Invicta Plastics Ltd., Leicester, UK) and weight was measured to the nearest 0.1 kg (with shoes and bulky clothing removed) using an electronic scale (Heine S-7307; Heine, NH, USA). Serum 25-hydroxyvitamin D level was measured using direct competitive chemiluminescent immunoassays (DiaSorin Inc., Stillwater, MN, USA). Energy intake was computed based on the DQES v2 using the Australian NUTTAB 95 database [13].

### 2.8. Statistical Analysis

Baseline characteristics were presented using means with standard deviation, proportions, and medians with quartile when appropriate. Student’s t-tests were used when comparing the outcomes at different follow-ups.

Associations between dietary macroelements and knee structure, OA symptoms, quality of life, and OA comorbid conditions over the study period were estimated using mixed-effects linear regression model, which allows for adjusting repeated measures at each follow-up (months 3, 6, 12, and 24) and protecting against bias for missing data [21]. In these models, dietary macroelements, age, sex, BMI, serum 25-hydroxyvitamin D level, energy intake, education, work status, and work type at baseline were entered as fixed effects, and individual participant identification was treated as random intercepts. As follow-up time points were not interacted with dietary macroelements, they were adjusted as fixed effects in the mixed-effects models. The adjusted associations between dietary macroelements and OA-related outcomes were expressed as maximum-likelihood estimated slope coefficients. The normality of model residuals and linearity trend were routinely checked. Statistical interactions between dietary macroelements and sex were assessed.

All statistical analyses were performed using Stata version 15.0 for Windows (StataCorp., College Station, TX, USA). A *p* value less than 0.05 (two tailed) was regarded as statistically significant.

## 3. Results

Characteristics of the participants at baseline are presented in Table 1. The average levels of dietary macroelements were 0.91 g/day for calcium, 0.30 g/day for magnesium, 2.86 g/day for potassium, and 1.53 g/day for phosphorus. As regard the outcomes with follow-ups, outcomes between adjacent visits were compared using Student’s unpaired t-test (Supplementary Table S1). The difference in total volume between baseline and 24-month follow-up (*p* = 0.003), in total cartilage defect score between baseline and 24-month follow-up (*p* = 0.044), and in WOMAC score between baseline and 3-month follow-up (*p* < 0.001) were significant (Supplementary Table S1); other differences between adjacent visits were not evident.

None of the four dietary macroelements were significantly associated with any of the knee joint structures including total cartilage volume, total cartilage defect, total BML, and total effusion-synovitis volume (Table 2). Statistical interactions between dietary macroelements and sex on knee joint structures were not significant (*p* > 0.1).

For knee OA symptoms, both dietary magnesium and potassium were significantly and negatively associated with total WOMAC (β = −753.93 for magnesium and −87.72 for potassium), WOMAC joint stiffness (β = −76.68 for magnesium and −9.62 for potassium), and WOMAC physical dysfunction (β = −592.18 for magnesium and −64.64 for potassium), but not significantly associated with WOMAC knee pain (Table 3). There was no significant association of dietary calcium and phosphorus with total WOMAC and any of the three WOMAC sub-scores (Table 3). Statistical interactions between dietary macroelements and sex were not significant (*p* > 0.1).

Magnesium intake had significantly negative associations with total AQoL (β = −7.61; 95% CI = −12.23, −2.99), AQoL social relationships (β = −2.75; 95% CI = (−4.38, −1.12), AQoL physical senses (β = −1.71; 95% CI = −3.11, −0.30), and AQoL psychological well-being (β = −2.52; 95% CI = −4.69, −0.35), but no significant association with independent living (Table 4). The associations of potassium intake with AQoL were similar to that of magnesium intake, except that the potassium intake was not significantly associated with AQoL physical senses. Both calcium and phosphorus were not significantly associated with total AQoL and any of the AQoL sub-scores (Table 4). Statistical interactions between dietary macroelements and sex were not significant (*p* > 0.1).

For OA comorbid conditions, dietary calcium had no significant association with lower limb muscle strength and PHQ-9 (Table 5). Dietary magnesium and potassium were positively associated with lower limb muscle strength (β = 66.50 for magnesium and 8.42 for potassium), and negatively associated with PHQ-9 (β = −7.52 for magnesium and −0.82 for potassium) (Table 5). Dietary phosphorus had no significant association with lower limb muscle strength but was positive association with lower limb muscle strength (Table 5). Statistical interactions between dietary macroelements and sex were not significant (*p* > 0.1).

## 4. Discussion

To the best of our knowledge, this is the first epidemiological study to comprehensively examine the longitudinal associations of dietary macroelements with knee joint structures, symptoms, quality of life, and comorbid conditions in symptomatic knee OA patients. We found that magnesium and potassium intake were associated with milder OA symptoms, better quality of life, and less comorbid conditions, while calcium and phosphorus intake were largely not associated with these outcomes in patients with knee OA. However, as an exploratory study, these results should be treated with caution and need to be confirmed by further experimental and clinical studies.

Calcium is an essential nutrient that plays a key role in the musculoskeletal system. However, studies about the relationship between calcium and OA are limited. To our knowledge, no data illustrate the association of calcium intake with OA to date. A few studies investigating the relevance of serum calcium and OA have shown inconsistent results. A cross-sectional study reported that the serum calcium level was inversely associated with radiographic knee OA [2]. Similarly, inverse causal associations of serum calcium level with overall OA, knee OA, and hip OA were suggested by a Mendelian randomization study [22]. Furthermore, an animal study found that oral administration of calcium gluconate had an OA-protective effect in rats, which was hypothesized to be due to anti-inflammatory activity of calcium salt [23]. However, there were also four studies that failed to find an association between serum/plasma calcium and OA [24,25,26,27]. In our study, calcium intake was not associated with the OA-related outcomes. In fact, calcium and magnesium antagonize each other in various physiological activities [2]. Competition for serum concentration may also exist between the two macroelements as they compete for intestinal absorption and a high calcium intake leads to increased urine excretion of magnesium [2]. Considering the significant associations of dietary magnesium with OA-related outcomes found in this study, the negative findings of associations between dietary calcium and knee OA-related outcomes seem reasonable. However, whether OA can benefit from higher calcium intake needs to be confirmed by further studies. 

Magnesium is one of the most important micronutrients for human health [28]. It influences inflammatory cytokine levels and acts as a pain mediator, as well as affecting muscle function [27]. Emerging evidence indicates that magnesium deficiency had a strong relationship with OA [29]. Studies reported that both dietary intake and serum concentration of magnesium have inverse relationships with radiographic knee OA [28,30]. Low magnesium intake was associated with worse knee cartilage architecture in subjects at risk of knee OA and increased knee pain in subjects with radiographic knee OA [31,32]. In animal experiments, intra-articular injection of magnesium salts can attenuate disease progression in rat OA models [33,34]. This could be explained by (1) the promotion of cartilage matrix synthesis and the suppression of synovial inflammation [33]; and (2) the attenuation of chondrocyte apoptosis and the reduction in nociception [34]. Of note, there are also studies that did not find a relationship between magnesium intake and knee OA [3,35]. Our study found higher magnesium intake was associated with fewer OA symptoms, higher quality of life, and milder comorbid conditions in knee OA patients, which added evidence supporting the important roles of magnesium in OA.

Potassium is an essential mineral that plays vital roles in nerve transmission, muscle contractions, regulation of blood pressure, and maintenance of the integrity of the skeleton [36]. Evidence suggested that low potassium intake was associated with diseases such as hypertension, cardiovascular disease, and osteoporosis [37]. However, to the best of our knowledge, there were no studies reporting the relationship between dietary potassium and OA. Only one study suggested that potassium-based intra-articular injection could be suitable for OA treatment. In this in vitro study, 80 mM K+ gluconate could suppress proinflammatory macrophage activation by driving macrophage polarization toward an anti-inflammatory phenotype [38]. Our study, for the first time, illustrated associations of dietary potassium with OA-related outcomes, which suggested a potential protective effect of dietary potassium on OA. More experimental studies are needed to elucidate the underlying mechanisms.

Phosphorus is one of the essential elements of the human body and is required for a diverse range of processes, such as ATP synthesis, signal transduction, and bone mineralization [39]. There was no study investigating the association between dietary phosphorus and OA. Our study found that dietary phosphorus was significantly associated only with greater lower limb muscle strength in knee OA. This significant association could be explained by the fact that phosphorus is required for maintaining muscle function as it is a substrate for ATP and creatine phosphate synthesis [39]. In fact, both low and high extracellular phosphorus is associated with adverse health outcomes, and phosphorus consumption in Western society exceeds that needed as dietary phosphorus is plentiful in the Western diet for food preservation [39]. Therefore, the only significant association found between dietary phosphate and OA-related outcomes should be treated with caution and validated by further study.

In summary, this study suggested that high magnesium and potassium intake may have protective effects in knee OA. Magnesium and potassium intake is reduced in modern society due to food processing, and the under-consuming of the two dietary nutrients nowadays has drawn public health concerns [36,40]. Of note, though the associations of magnesium and potassium with WOMAC score were statistically significant, changes would only be clinically meaningful if the WOMAC score decrease by 77.04 given the baseline WOMAC of 642.00 as the minimum clinically important difference of WOMAC score in patients with knee OA is reportedly 12% of the baseline score [40]. Given the β in our study was −753.93 g/day for magnesium and −87.72 g/day for potassium, the changes of WOMAC scores would only be clinical meaningful if magnesium increased by 0.10 g/day and potassium increased by 0.88 g/day. Based on the interquartile range of magnesium and potassium in our study of 0.13 g/day and 1.02 g/day, respectively, supplementation of the two nutrients could be a useful and feasible strategy for knee OA management. However, which particular subject demographic may benefit most from supplementation of the two nutrients and at what dosages are difficult to determine because the current evidence comes from observational studies. Thus, rigorously designed randomized controlled trials are desirable. Notably, our study did not find significant associations of dietary magnesium and potassium with knee OA structural features, suggesting that the potential beneficial effects of the nutrients on knee OA could be mainly related to symptoms; however, the results should be verified by further studies.

There are several potential limitations in this study. First, this was an exploratory study that can only generate hypotheses; therefore, the results should be interpreted with caution and verified by further confirmatory studies. Second, it was a post-hoc analysis of a randomized controlled trial with strict inclusion and exclusion criteria; therefore, the results may not be generalizable to the general knee OA population. Third, some potential confounding factors may not be adjusted, and with the limited data, it may not be possible to adjust for all confounding factors for each outcome. Last, the sample size was modest, and it is possible that with a larger sample size more significant associations can be detected.

## 5. Conclusions

Higher dietary magnesium and potassium intake are associated with fewer OA symptoms, higher quality of life, and milder comorbid conditions in patients with knee OA, suggesting that dietary magnesium and potassium may have beneficial effects on knee OA and could be used for knee OA management.

## Figures and Tables

**Table 1:** Characteristics of participants at baseline.

Characteristic (*n* = 392)	
Age (years) ^a^	63.26 (7.11)
Male (%) ^c^	50.51
Height (cm) ^a^	168.36 (9.65)
Weight (kg) ^a^	83.87 (15.76)
BMI (kg/m^2^) ^a^	29.56 (5.04)
25-hydroxyvitamin D (nmol/L) ^a^	43.84 (12.07)
Energy intake (kj/day) ^a^	7321.78 (2733.29)
Education ^c^	
Didn’t finish high school (%)	12.34
Finished high school (%)	25.19
Trade/apprenticeship (%)	12.34
Certificate/diploma (%)	26.99
Bachelor degree or higher (%)	23.14
Work status ^c^	
Full-time employed (%)	28.02
Part-time/casual employment (%)	21.85
Unemployed (%)	1.29
Home duties (%)	1.54
Retired (%)	46.27
Student (%)	0
Other (%)	1.03
Work type^c^	
Manual (%)	33.07
Office/professional (%)	66.41
Not applicable (%)	0.52
Total cartilage volume (cm^3^) ^a^	5.76 (1.65)
Total cartilage defect score ^a^	14.59 (4.01)
Total BML score ^b^	3.00 (1.00, 5.00)
Total effusion-synovitis volume (ml)^b^	5.25 (2.33, 10.68)
WOMAC ^b^	642.00 (327.50, 977.00)
AQoL ^a^	16.35 (2.90)
Lower limb muscle strength (kg) ^b^	60.00 (33.67, 96.67)
PHQ-9 ^b^	2.00 (0.00, 5.00)
Calcium (g/day) ^a^	0.91 (0.31)
Magnesium (g/day) ^a^	0.30 (0.10)
Potassium (g/day) ^a^	2.86(0.88)
Phosphorus (g/day) ^a^	1.53(0.52)

BMI: body mass index; BML: bone marrow lesion; WOMAC: Western Ontario and McMaster Universities; AQoL: Assessment of Quality of Life; PHQ-9: 9-items Patient Health Questionnaire. a demonstrated as mean (standard deviation); b demonstrated as median (25% percentile, 75% percentile); c demonstrated as percentage.

**Table 2:** Associations of macroelements with knee joint structures over 2 years.

	Calcium (g/Day)		Magnesium (g/Day)		Potassium (g/Day)		Phosphorus (g/Day)	
	β (95% CI)	*p* Value	β (95% CI)	*p* Value	β (95% CI)	*p* Value	β (95% CI)	*p* Value
Cartilage volume (cm^3^)	0.33 (−0.12, 0.77)	0.149	1.01 (−1.08, 3.10)	0.344	0.12 (−0.14, 0.38)	0.374	0.17 (−0.31, 0.65)	0.497
Cartilage defects	0.51 (−0.95, 1.98)	0.491	0.77 (−6.10, 7.63)	0.827	0.16 (−0.69, 1.02)	0.705	0.62 (−0.96, 2.20)	0.439
BML	−0.04 (−1.27, 1.20)	0.953	2.39 (−3.40, 8.19)	0.418	0.02 (−0.70, 0.74)	0.965	−0.12 (−1.46, 1.21)	0.856
Effusion-synovitis volume (ml)	0.16 (−3.08, 3.40)	0.924	−2.57 (−17.73, 12.59)	0.739	−0.84 (−2.72, 1.04)	0.383	0.26 (−3.23, 3.75)	0.882

BML: bone marrow lesion.

**Table 3:** Associations of macroelements with WOMAC score over 2 years.

	Calcium (g/Day)		Magnesium (g/Day)		Potassium (g/Day)		Phosphorus (g/Day)	
	β (95% CI)	*p* Value	β (95% CI)	*p* Value	β (95% CI)	*p* Value	β (95% CI)	*p* Value
Total WOMAC score	−44.45 (−174.77, 85.87)	0.504	**−753.93 (−1355.89, −151.97)**	**0.014**	**−87.72 (−163.18, −12.25)**	**0.023**	−102.03 (−242.04, 37.97)	0.153
Pain	−16.67 (−42.34, 9.00)	0.203	−80.30 (−199.71, 39.10)	0.187	−13.00 (−27.95, 1.95)	0.088	−16.27 (−43.88, 11.34)	0.248
Stiffness	−3.91 (−16.64, 8.83)	0.548	**−76.68 (−135.54, −17.83)**	**0.011**	**−9.62 (−16.99, −2.25)**	**0.011**	−7.81 (−21.50, 5.88)	0.264
Physical dysfunction	−23.40 (−119.51, 72.71)	0.633	**−592.18 (−1035.56, −148.81)**	**0.009**	**−64.64 (−120.27, −9.01)**	**0.023**	−77.09 (−180.32, 26.14)	0.143

WOMAC: Western Ontario and McMaster Universities. Data in bold denote statistically significant results.

**Table 4:** Associations of macroelements with AQoL over 2 years.

	Calcium (g/Day)		Magnesium (g/Day)		Potassium (g/Day)		Phosphorus (g/Day)	
	β (95% CI)	*p* Value	β (95% CI)	*p* Value	β (95% CI)	*p* Value	β (95% CI)	*p* Value
Total AQoL score	−1.00 (−2.02, 0.01)	0.053	**−7.61 (−12.23, −2.99)**	**0.001**	**−0.87 (−1.45, −0.29)**	**0.003**	−0.98 (−2.07, 0.10)	0.075
Independent living	−0.16 (−0.39, 0.09)	0.214	−0.57 (−1.71, 0.57)	0.329	−0.03 (−0.17, 0.11)	0.691	−0.05 (−0.31, 0.22)	0.721
Social relationships	−0.26 (−0.62, 0.96)	0.151	**−2.75 (−4.38, −1.12)**	**0.001**	**−0.41 (−0.61, −0.21)**	**<0.001**	−0.20 (−0.58, 0.18)	0.301
Physical senses	−0.18 (−0.49, 0.12)	0.236	**−1.71 (−3.11, −0.30)**	**0.017**	−0.10 (−0.28, 0.08)	0.263	−0.27 (−0.59, 0.06)	0.108
Psychological well-being	−0.42 (−0.89, 0.04)	0.076	**−2.52 (−4.69, −0.35)**	**0.023**	**−0.34 (−0.61, −0.07)**	**0.013**	−0.45 (−0.95, 0.05)	0.080

AQoL: Assessment of Quality of Life. Data in bold denote statistically significant results.

**Table 5:** Associations of macroelements with OA comorbid conditions over 2 years.

	Calcium (g/Day)		Magnesium (g/Day)		Potassium (g/Day)		Phosphorus (g/Day)	
	β (95% CI)	*p* Value	β (95% CI)	*p* Value	β (95% CI)	*p* Value	β (95% CI)	*p* Value
Lower limb muscle strength (kg)	7.32 (−3.01, 17.65)	0.165	**66.79 (18.47, 115.11)**	**0.007**	**8.35 (2.37, 14.33)**	**0.006**	**11.33 (0.18, 22.48)**	**0.046**
PHQ−9	−0.83 (−1.99, 0.32)	0.156	**−7.52 (−12.88, −2.16)**	**0.006**	**−0.82 (−1.49, −0.15)**	**0.017**	−0.99 (−2.23, −0.25)	0.117

PHQ-9: 9-items Patient Health Questionnaire; data in bold denote statistically significant results.

## Data Availability

Data can be obtained from the corresponding authors upon reasonable request.

## Supplementary Materials

The following supporting information can be downloaded at: https://www.mdpi.com/article/10.3390/nu14173576/s1, Table S1: Means and standard deviations of the outcomes at baseline and follow-ups.
