# Supplementary material for: Associations of Dietary Macroelements with Knee Joint Structures, Symptoms, Quality of Life, and Comorbid Conditions in People with Symptomatic Knee Osteoarthritis
Source: Nutrients. 2022 Aug 30;14(17):3576. doi: 10.3390/nu14173576 (PMC9460692; doi:10.3390/nu14173576)
Supplement: Supplementary file 1 [file nutrients-14-03576-s001.zip › nutrients-1814635-supplementary.pdf]

Supplementary Table S1. Means and standard deviations of the outcomes at baseline and follow-ups.

Outcomes	Values at follow-up time points (mean±standard deviation)				
	**Baseline (n)**	**3-month (n)**	**6-month (n)**	**12-month (n)**	**24-month (n)**
**Total cartilage volume (cm^3)**	5.76±1.65 (388)	-	-	-	5.39±1.61* (341)
**Total cartilage defect score**	14.59±4.01 (388)	-	-	-	15.20±4.12* (343)
**Total BML score**	3.40±3.28 (388)	-	-	-	3.41±3.63 (342)
**Total effusion-synovitis volume (ml)**	8.15±8.66 (388)	-	-	-	9.21±10.66 (338)
**WOMAC**	670.59±409.73 (388)	560.77±423.09* (367)	523.56±406.02 (363)	504.91±437.54 (340)	467.54±426.46 (331)
**AQoL**	(16.35±2.90) (385)	-	(16.33±2.97) (363)	(16.25±2.99) (338)	(16.11±2.88) (320)
**Lower limb muscle strength (kg)**	67.00±40.89 (383)	-	71.50±40.14 (326)	73.86±40.83 (296)	70.31±40.73 (250)
**PHQ-9**	3.19±4.05 (380)	2.99±3.27 (364)	2.88±3.40 (357)	2.91±3.66 (336)	2.80±3.42 (321)

* Using Student's unpaired t test, differences were significant in total volume between baseline and 24-month follow-up (*P*=0.003); in total cartilage

defect score between baseline and 24-month follow-up (*P*=0.044); in WOMAC score between baseline and 3-month follow-up (*P*<0.001).

WOMAC: Western Ontario and McMaster Universities; AQoL: Assessment of Quality of Life; PHQ-9: 9-items Patient Health Questionnaire.
